# Privatization is not an answer to health care access problems, increased public funding is

**DOI:** 10.3747/co.v16i2.351

**Published:** 2009-03

**Authors:** Ervin B. Podgorsak

**Affiliations:** Ervin B. Podgorsak is professor of Medical Physics at McGill University in Montreal. He was awarded the 2008 Gold Medal from the Canadian Organization of Medical Physics, and this article is an excerpt from the acceptance speech he gave in Quebec City on June 27, 2008

The most important characteristics of a health care system are its *quality, access,* and *cost*. The Canadian health care system is of high quality; however, access to it is definitely problematic, and arguably, its cost is high.

The obvious solution to access problems is increased public funding by 15%–20% above the current level. Universal and timely access to health care in Canada is a chronic problem, resulting in waiting lists not only for elective surgical procedures but often also for essential diagnostic procedures such as computed tomography (ct) and magnetic resonance imaging (mri) examinations and for emergency treatment such as cancer radiotherapy. While problems with access to Canadian health care are real and serious, the causes of these problems are shrouded in many myths and misconceptions, especially in relation to the merits of public administration and in the perception of high cost and inefficiency.

The public administration of the Canadian health care service is one of the most cherished defining characteristics of Canada, yet many special interest groups are touting privatization as the only viable solution to the current health care access problems. Privately run health care can attain high quality standards and provide excellent access to insured patients as well as to those who are willing to pay for services. However, it also results in a U.S.-type two-tiered and socially unjust medical system in which access to health care depends on patients’ ability to pay for services rather than on the need for them.

The vast majority of Canadians would not want to emulate the privatized U.S. health care system; yet, health care privatization is slowly creeping into Canada. This is happening despite the principle of public administration that is enshrined in the *Canada Health Act*, but unfortunately is poorly enforced by the federal government.

The Canadian public is constantly bombarded with claims alluding to excessive cost and poor efficiency of the Canadian health care system. However, it is actually easy to show that Canadian governments, despite their protestations to the contrary, do not spend enough for health care, and this shortfall is the main reason for the current serious problems with access to health care for all Canadians.

We often hear that health care expenditures are out of control, having increased more than tenfold from 1975 to 2005. Yet, once one accounts for the increase in the consumer price index by a factor of 3.7 and the increase in the population by a factor of 1.4 for the same period, one finds that health care costs effectively increased only by a factor of 2 in 30 years. Considering that, in 1975, ct scanners had just appeared, there were no mri machines yet, computerization and the Internet were in the distant future, and many of today’s standard diagnostic and therapeutic procedures were still to be discovered or developed, doubling of health care costs in 30 years is certainly not excessive, especially if we compare it to the doubling in the cost of oil during the past year.

The Organization for Economic Cooperation and Development (oecd), a closed club of 30 countries, most of them developed, provides useful statistics on the development of individual member states as well as averages for the whole group. Canada is an oecd country and its performance in terms of health care indicators ranges from slightly above average in life expectancy and infant mortality to scandalously below average in access to physicians and such high technology diagnostic equipment as mri and ct scanners.

To solve the health care access problem in Canada, no elaborate and costly studies, committees, or commissions are required. What we need are reasonable and achievable standards and goals for the Canadian health care system and adequate government support to meet the standards and achieve the goals. **For non-monetary health indicators, matching the** **oecd** **average should be the minimum standard, and exceeding the** **oecd** **average should be the goal**.

Unfortunately, Canadian politicians zero in on the cost rather than the performance of our health care system. Canada spends 10% of its gross national product (gnp) on health care as compared with a 9% average for the oecd countries. However, several oecd countries, at 11%, rank above Canada, and the United States is in a league of its own at 16%. As a society, Canada decided to give better-than-oecd-average remuneration to its health care workers, and this choice invariably will result in a higher-than-average gnp cost percentage. However, rationing access to health services to compensate for the higher remuneration of health care workers is short-sighted and not in the best interest of Canadians. Yet, this is exactly what Canadian politicians are doing by keeping the gnp percentage spent on health care close to the oecd average, thereby throwing all the important indicators that control access to health care services shamefully below the oecd average. This misguided policy then results in waiting lists, delayed or denied diagnostic and therapeutic procedures, frustration with the health care system, and a stampede to undesirable privatization. There is nothing magic about the current 10% of gnp level; Canada can afford to spend 11% or even 12% of gnp to bring the health care access problem under control.

A closer look at the current situation reveals that, to attain the oecd average, Canada would need to double the number of its mri machines from 162 to 324 and to increase its number of ct scanners by 324 from the current number of 356 at a one-time cost of $1 billion. The 500 new imaging machines would require 1200 new technologists—staff that are currently not available in Canada—and the operating expenses for maintenance and staff would be about $200 million annually.

The situation is just as bleak when one considers the number of physicians practicing in Canada. To reach the oecd average of 3 physicians per 1000 population from the current level of 2.1, Canada would need to add 30,000 new physicians to its current 70,000 an extremely difficult proposition, considering that the 17 Canadian medical schools produce only about 2500 new physicians per year and that this number barely compensates for retirement and emigration of physicians.

Allowing Canada to languish significantly below oecd averages in access to health care is a disservice to all Canadians. It is obvious that governments must stop obsessing about cost and switch their priority to providing sufficient funding to ensure high quality health services without waiting lists. The federal government, through its *Canada Health Act*, has the means and obligation not only to set simple and clear standards and goals, but also to produce most of the required cash.

When the federal government introduced the public health care system in the late 1960s, its cost-sharing formula with the provinces was 50%–50%; however, with passing decades the federal share dwindled to the current level of 25%. In an era of federal budget surpluses, the expectation that the federal government improve this obvious “fiscal imbalance” in health care financing toward the provinces seems reasonable, realistic, and urgent. Rather than insisting on 10% or less of the gnp for health care costs, the Canadian federal and provincial governments should provide whatever it takes to get all non-monetary health care indicators above the oecd average. Canadian health care access problems can be remedied by a budget increase of 15%–20%. Canada can afford this, Canadians deserve this, and the governments should finally recognize this with extraordinary funding initiatives. Of course, the staff shortages cannot be solved overnight, but increased funding would be an important step in the right direction.

